# Oxycodone vs. sufentanil combined with quadratus lumborum block vs. transverse abdominis plane block in laparoscopic major gastrointestinal surgery: A randomized factorial trial protocol

**DOI:** 10.1016/j.heliyon.2024.e36186

**Published:** 2024-08-15

**Authors:** Guo-wang Yang, Min-yuan Zhuang, Hai-jing Shi, Xiao-yang Song, Hong Liu, Fu-hai Ji, Ke Peng

**Affiliations:** a Department of Anesthesiology, The First Affiliated Hospital of Soochow University, Suzhou, Jiangsu, 215006, China; b Institute of Anesthesiology, Soochow University, Suzhou, Jiangsu, 215006, China; c Department of Anesthesiology, Suzhou Municipal Hospital, The Affiliated Suzhou Hospital of Nanjing Medical University, Suzhou, Jiangsu, 215006, China; d Department of Anesthesiology and Pain Medicine, University of California Davis Health, Sacramento, CA, 95817, USA

**Keywords:** Oxycodone, Patient-controlled analgesia, Quadratus lumborum block, Transverse abdominis plane block, Quality of recovery

## Abstract

**Background:**

Multimodal analgesia plays a key role in enhanced recovery after surgery. Herein, we describe a trial protocol investigating the effects of oxycodone-vs. sufentanil-based patient-controlled analgesia in combination with quadratus lumborum block (QLB) vs. transverse abdominis plane block (TAPB) on quality of recovery following major laparoscopic gastrointestinal surgery.

**Methods:**

and analysis: This is a prospective, randomized, controlled clinical trial with a 2 × 2 factorial design. A total of 120 adult patients undergoing laparoscopic major gastrointestinal surgery will be randomized, in a 1:1:1:1 ratio, to receive one of two patient-controlled analgesia regimens (based on oxycodone or sufentanil) and one of two regional blocks (QLB or TAPB). The primary outcome measure of this trial is the quality of recovery at 24 h after surgery, assessed using the 15-item quality of recovery (QoR-15) scale. The secondary outcomes include QoR-15 scores at 48 and 72 h after surgery; visceral and incisional pain at rest and while coughing at 1, 6, 24 and 48 h postoperatively; analgesic consumption within 0–24 h and 24–48 h postoperatively; need for rescue analgesia; postoperative flatus time; postoperative adverse events (sedation, nausea and vomiting, use of antiemetics, respiratory depression, and dizziness); and length of postoperative hospital stay.

**Discussion:**

The results of this trial will provide evidence for the optimal multimodal analgesic strategy to improve the quality of recovery for patients undergoing laparoscopic major gastrointestinal surgery.

**Trial registration:**

This trial was registered at the Chinese Clinical Trial Registry (www.chictr.org.cn, identifier: ChiCTR2400080766).

## 1 Introduction

According to the Global Cancer Statistics 2020, gastric and colorectal cancer are among the top five causes of cancer death worldwide, leading to an estimated 1.7 million deaths [1]. Laparoscopic surgery has been widely performed for patients with gastrointestinal cancer [[2], [3], [4], [5]]. After these laparoscopic procedures, many patients often experience significant pain, especially visceral pain, which increases the risks of postoperative morbidities and comprises the quality of recovery after surgery.

Multimodal analgesic strategy is primarily intended to enhance analgesia and reduce opioid-related adverse events [6,7]. These improvements contribute to better postoperative recovery. Sufentanil is a classic opioid agent widely used in various surgical procedures, while oxycodone is a semi-synthetic opioid analgesic that can relieve both visceral and somatic pain owing to its action on both the κ and μ opioid receptors [[8], [9], [10]]. As an important element of multimodal analgesia, regional blocks such as quadratus lumborum block (QLB) and transverse abdominis plane (TAPB) have been applied in abdominal surgery [11,12]. Compared with TAPB, the use of QLB may lead to a better relief of postoperative visceral and somatic pain [13,14]. However, the effects of oxycodone or QLB and their combination on the quality of recovery after laparoscopic gastrointestinal surgery are yet to be determined.

Therefore, this randomized controlled factorial trial is designed to compare oxycodone-with sufentanil-based postoperative analgesia combined with QLB vs. TAPB among patients undergoing laparoscopic major gastrointestinal surgery. The primary outcome measure is patients' quality of recovery at 24 h after surgery. We hypothesize that the use of oxycodone and QLB would enhance recovery after these laparoscopic surgical procedures.

## 2 Methods

### 2.1 Trial design

This prospective, randomized, controlled, factorial clinical trial is conducted at the First Affiliated Hospital of Soochow University, Suzhou, Jiangsu, China. In this hospital, the annual volume of laparoscopic gastrointestinal surgery is approximately 6000. Patient recruitment started on March 1, 2024. This trial is ongoing at the time of this protocol submission. We plan to recruit a total of 120 eligible patients before March 31, 2025.

**Fig. 1:**
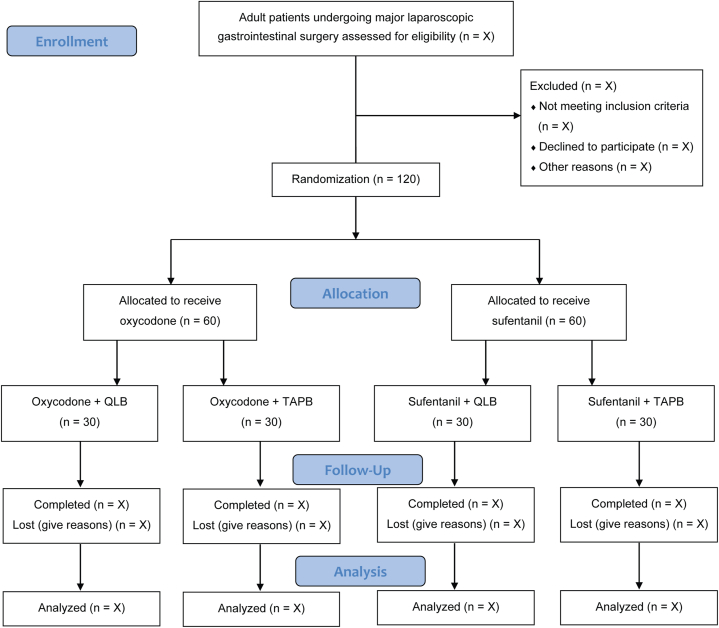
Study flow diagram.

With the 2 × 2 factorial design, we are able to evaluate the main effects of two treatments in one study and explore their potential interaction [[15], [16], [17]]. In this trial, one treatment is patient-controlled analgesia (using oxycodone vs. sufentanil), and the other treatment is regional blocks (QLB vs. TAPB). Thus, there are 4 treatment combinations in this study: oxycodone-QLB, oxycodone-TAPB, sufentanil-QLB, and sufentanil-TAPB. The flowchart of this trial is presented in Fig. 1.

### 2.2 Eligibility criteria

Adult patients with ASA physical status I–III who are scheduled to undergo laparoscopic major gastrointestinal surgery (an estimated surgical time ≥2 h) are eligible for inclusion.

Exclusion criteria include (1) unplanned or emergency surgery; (2) body mass index ≥35 kg/m^2^; (3) allergy to drugs used in this study; (4) serious preoperative diseases (such as myocardial infarction, heart failure, respiratory failure, cerebral hemorrhage, stroke, Child-Pugh C grade, renal replacement therapy, Parkinson's disease, Alzheimer's disease); (5) antipsychotic use, alcoholism, long-term use of opioids or other analgesic drugs; or (6) inability to communicate or understand the rating scales for postoperative recovery and pain.

### 2.3 Randomization and blinding

A researcher not involved in patient enrollment, data collection, perioperative care, or outcome assessment utilizes the online randomization tool (Sealed Envelope) to generate a random sequence with a 1:1:1:1 ratio and a block size of 8. The random numbers are concealed in opaque sealed envelopes. Approximately 0.5–1 h before anesthesia, a research nurse unaware of the randomization details open the envelopes to assign patients into the study groups, and prepares oxycodone and sufentanil in identical syringes labelled with study codes only. Responsible anesthesiologists are blinded to the use of oxycodone and sufentanil, but not to QLB and TAPB. All patients and two outcome investigators (HJS and MYZ) will remain blinded to the group allocation. Specifically, they will neither access anesthesia records nor participate in patient care.

### 2.4 Anesthesia and study interventions

In the operating room, all patients will be monitored with electrocardiography, cuff blood pressure, pulse oximetry, and nasal temperature. Artery blood pressure will be continuously monitored via radial artery canulation, and anesthesia depth will be monitored using bispectral index (BIS).

For all patients, propofol 1.5–2 mg/kg and sufentanil 0.2 μg/kg will be used to induce general anesthesia, and cisatracurium 0.2 mg/kg will be given for tracheal intubation. Anesthesia will be maintained using sevoflurane, adjusted to BIS values within 40–60. Remifentanil will be infused at 0.05–0.2 μg/kg/min and cisatracurium 0.1 mg/kg will be administered when needed. Ventilation will be adjusted to peripheral oxygen saturation ≥95 % and end-tidal carbon dioxide within 35–45 mmHg. Patients will receive dexamethasone 5 mg and palonosetron 0.25 mg for postoperative nausea and vomiting (PONV) prophylaxis.

All patients will receive multimodal analgesia including the following components: QLB or TAPB (ultrasound-guided bilateral blocks with 0.375 % ropivacaine 20 mL at each side after intubation and before skin incision), dexmedetomidine (0.4 μg/kg/h after intubation until 30 min before the end of surgery), flurbiprofen axetil (50 mg at the end of surgery), and oxycodone- or sufentanil-based patient-controlled analgesia (PCA) during the first two postoperative days. Patients will receive a loading dose of oxycodone 0.2 mg/kg or sufentanil 0.2 μg/kg before skin closure, followed by oxycodone- or sufentanil-based PCA (oxycodone 2 mg or sufentanil 2 μg at each bolus with a lockout time of 5 min). During the postoperative period, pain with intensity ≥4 points on a numerical rating scale (NRS, 0–10; 0 = no pain and 10 = the worst pain imaginable) will be treated with a bolus of oxycodone or sufentanil through the PAC system. Hypertension (an increase in mean arterial pressure >30 % of baseline value), hypotension (a decrease in mean arterial pressure >30 % of baseline value), tachycardia (heart rate >100 beats/min), and bradycardia (heart rate <45 beats/min) will be treated, and other anesthetic care will be provided at the discretion of responsible anesthesiologists.

### 2.5 Study outcomes

The primary outcome is the quality of recovery at 24 h after surgery, assessed using the 15-item quality of recovery (QoR-15) scale (0–150 points; a higher score indicating a better recovery quality) [[18], [19], [20]]. Patients will be left alone to complete the QoR-15 questionnaire on their own. Based on the literature, a difference of 6.0 in QoR-15 scores denotes a minimal clinically important difference [21,22].

The secondary outcomes include QoR-15 scores at 48 and 72 h after surgery; visceral and incisional NRS pain scores at rest and while coughing at 1, 6, 24 and 48 h postoperatively; analgesic consumption within 0–24 h and 24–48 h postoperatively; need for rescue analgesia; postoperative flatus time; postoperative adverse events (sedation, PONV, use of antiemetics, respiratory depression, and dizziness); and length of postoperative hospital stay.

### 2.6 Data collection and monitoring

The schedule of this trial is presented in Table 1. Demographic and baseline characteristic data will be collected during preoperative ward visits. Intraoperative data will be extracted from electronic anesthesia records. Postoperative outcome data will be recorded by the blinded assessors (HJS and MYZ). All data will be documented in case report forms (Supplemental file 1) and then registered in an electronic database. The principal investigator (KP) will check the accuracy and completeness of all data. In our institution, an independent data monitoring committee will perform an ongoing review of the study process. After trial completion, a statistician will access the dataset without allocation details or personally identifiable information for data analysis based on a predefined statistical plan.

**Table 1:** Schedule of enrollment, interventions, and assessment.

**Timepoint**	Study Period						
	Enrollment	Allocation	Post-allocation				Close-out
	*Pre-anesthesia visit*	*Prior to anesthesia*	*PACU*	*Postoperative* *24h*	*Postoperative* *48h*	*Postoperative* *72h*	*Hospital discharge*
**Enrollment**							
Eligibility criteria	×						
Written informed consent	×						
Demographic data	×						
Baseline characteristics	×						
Randomization		×					
Allocation		×					
Interventions rowhead							
Oxycodone + QLB		×					
Oxycodone + TAPB		×					
Sufentanil + QLB		×					
Sufentanil + TAPB		×					
Assessment rowhead							
QoR-15 scores				×	×	×	
Visceral pain scores			×	×	×		
Incision pain scores			×	×	×		
Analgesic consumption				×	×		
Rescue analgesia				×	×		
Flatus time				×	×	×	×
Adverse events			×	×	×		
Postoperative stay							×

According to SPIRIT 2013 statement of defining standard protocol items for clinical trials.

### 2.7 Sample size calculation

According to our pilot observation, the mean ± standard deviation (SD) QoR-15 score at 24 h after laparoscopic major gastrointestinal surgery was 99 ± 10 in patients receiving patient-controlled sufentanil and TAPB (n = 20) [23]. Considering the minimal clinically important difference for the QoR-15 scale, we expect that the use of oxycodone or QLB would lead to an increase of 6 points in the QoR-15 scores [21]. With an α level of 0.025 and a power of 80 %, a total of 110 patients will be required for the main effects of oxycodone vs. sufentanil and QLB vs. TAPB (n = 55 in each group). To account for possible dropouts, we set the sample size to 120 (n = 60 for each comparison in the main effects; n = 30 in each of the four study groups). The sample size was calculated using the PASS software (version 15, NCSS, LLC. Kaysville, Utah, USA).

### 2.8 Statistical analysis

Data will be reported as means with SDs, medians with interquartile ranges, or numbers with percentages, depending on data type and distribution. In this factorial trial, an interaction analysis will be conducted using the two-way analysis of variance (ANOVA) with 24-h QoR-15 scores as the outcome variable to assess whether there is a significant interaction between the two study treatments. If no significant interaction is detected, the treatment effects can be summarized as main effects (oxycodone vs. sufentanil and QLB vs. TAPB); otherwise, each treatment effect should be evaluated within the levels of the other treatment.

For the main effects, groups will be compared using the unpaired *t*-test, Mann-Whitney rank-sum test, χ^2^ test, or Fisher's exact test, as appropriate. The study outcomes will be further analyzed using mean differences or relative risks with 95 % confidence intervals. An analysis across four factorial groups will be conducted using one-way ANOVA, Kruskal-Wallis test, or χ^2^ test, as appropriate. The primary analyses will be performed in the modified intention-to-treat population (patients after randomization and having outcome data available). Missing data will not be imputed. Because the primary outcome will be analyzed in two comparisons, the significance level is set at 0.025 for each treatment effect (i.e., 0.05/2 with the Bonferroni correction). The secondary outcomes will be regarded as exploratory with no corrections for multiple comparisons. All analyses will be performed using the SPSS software (version 25.0; IBM SPSS, Chicago, IL, USA).

## 3 Discussion

In this randomized controlled factorial trial, we aim to compare the effects of patient-controlled oxycodone vs. sufentanil combined with QLB vs. TAPB on the 24-h quality of recovery by using the QoR-15 scale in patients undergoing laparoscopic major gastrointestinal surgery. Based on the factorial design, we will explore whether there is a significant interaction between these two analgesic interventions. We will primarily assess the main effects of the treatments, and we will also conduct an analysis across four factorial groups. As far as we know, this is the first factorial trial on the role of patient-controlled analgesic strategies and regional blocks in patients recovering from laparoscopic major gastrointestinal surgery.

Perioperative medicine is the practice of patient-centered, multidisciplinary, and integrated medical care for surgical patients [24]. Anesthesiologists have special and rich experience in providing perioperative care, ensuring patient safety, and playing a leading role in the perioperative scenarios [25]. In perioperative medicine, the QoR-15 scale is widely used as a global measure of recovery for patients undergoing surgery [[26], [27], [28], [29], [30]]. This scale consists of 15 questions in 5 domains (i.e., physical independence, physical comfort, pain, psychological state, and emotional state), with a higher score suggesting a better quality of recovery [[18], [19], [20]]. Here, we focus on patients' recovery after surgery instead of individual indicators such as postoperative pain, analgesic consumption, or adverse events. By using the QoR-15 scale, we can get a comprehensive understanding of patient state after surgery from multiple dimensions.

This study has some limitations. First, the anesthesiologists are blinded to the patient-controlled analgesic regimens, but not blinded to the regional blocks. Next, we plan to include a total of 120 patients into four study groups. This is a relatively small sample size. This number of patients is based on the sample size calculation of the main effects, but it be insufficient for the interaction analysis.

This randomized factorial trial compares oxycodone-with sufentanil-based PCA combined with QLB vs. TAPB among patients undergoing laparoscopic major gastrointestinal surgery. With this trial, we will add to the evidence for an optimal multimodal analgesic strategy to improve the quality of recovery after these surgical procedures.

## 4 Ethics and dissemination

This trial was approved by the Ethics Committee of the First Affiliated Hospital of Soochow University on January 31, 2024 (no. 2024-039) (Supplemental file 2) and then registered at the Chinese Clinical Trial Registry (identifier: ChiCTR2400080766; available at: https://www.chictr.org.cn/showprojEN.html?proj=220080) on February 6, 2024. We conduct this study in accordance with the Declaration of Helsinki. All patients will provide written inform consent (Supplemental file 3). The original protocol submitted for ethical approval is available in Supplemental file 4. This protocol strictly follows the reporting guideline of Standard Protocol Items: Recommendations for Interventional Trials (Supplemental file 5) [31]. The results of this trial will be published in peer-reviewed journals. In addition, we plan to present this study at national and international academic conferences.

## Disclosure statement

No potential conflict of interest was reported by the authors.

## Funding

This study will be supported by the Suzhou Medical Health Science and Technology Innovation Project (SKY2022136) and Beijing Red Lilac Public Welfare Development Center Clinical Research Project (BJ-HDX-20211168-5). The funders have no role in the study design, data collection, data analysis, interpretation, or writing of this manuscript.

## Data sharing statement

No data was used for the research described in the article.

## Patient and public involvement

Patients and the public will not be involved in the design, recruitment, conduct, or report of the study. The study results will be disseminated to the participants via telephone or email.

## CRediT authorship contribution statement

**Guo-wang Yang:** Writing – original draft, Visualization, Validation, Software, Methodology, Investigation, Conceptualization. **Min-yuan Zhuang:** Writing – original draft, Visualization, Methodology, Investigation, Conceptualization. **Hai-jing Shi:** Writing – original draft, Validation, Methodology, Investigation, Conceptualization. **Xiao-yang Song:** Writing – original draft, Validation, Software, Methodology. **Hong Liu:** Writing – review & editing, Visualization, Methodology, Conceptualization. **Fu-hai Ji:** Writing – review & editing, Validation, Supervision, Methodology, Conceptualization. **Ke Peng:** Writing – review & editing, Visualization, Supervision, Resources, Methodology, Investigation, Funding acquisition, Conceptualization.

## Declaration of competing interest

The authors declare that they have no known competing financial interests or personal relationships that could have appeared to influence the work reported in this paper.
