# Supplementary material for: Oxycodone vs. sufentanil combined with quadratus lumborum block vs. transverse abdominis plane block in laparoscopic major gastrointestinal surgery: A randomized factorial trial protocol
Source: Heliyon. 2024 Aug 15;10(16):e36186. doi: 10.1016/j.heliyon.2024.e36186 (PMC11381733; doi:10.1016/j.heliyon.2024.e36186)
Supplement: Multimedia component 1 [file mmc1.pdf]

Case Report Form

Version Number: 1.0

Version Date: August 1, 2023

Random Number: _______________

I. Inclusion Criteria Checklist		
Inclusion Criteria<br>(Any column below must be "Yes", otherwise the patient cannot be included)	YES	NO
1. Age ≥ 18 years, sex not limited		
2. American Society of Anesthesiologists (ASA) I to III		
3. Scheduled for laparoscopic major gastrointestinal surgery under general anesthesia with an estimated surgery time ≥ 2 hours		
4. An estimated postoperative hospital stay ≥ 2 days		
5. Clear understanding of the study process and signing the informed consent form		
If any of the above questions are answered "No", the case cannot be included in the study.		
**II. Exclusion Criteria Checklist**		
Exclusion Criteria<br>(Any column below must be "No", otherwise the patient cannot be included)	YES	NO
1. Emergency surgery		
2. Body Mass Index (BMI) ≥ 35 kg/m^2		
3. Allergy to drugs used in the study		
4. Severe cardiopulmonary disease		
5. Severe cerebrovascular disease		
6. Severe liver and kidney diseases (Child-Pugh C, renal replacement therapy)		
7. Severe neurological and psychiatric diseases		
8. Alcohol abuse, long-term use of opioids or other analgesics		
9. Inability to understand postoperative recovery scale or pain score scale		
10. Hearing or language barriers that prevent communication		
11. Unwilling to use Patient-Controlled Intravenous Analgesia (PCIA)		
If any of the above questions are answered "Yes", the case cannot be included in the study.		

III. Baseline Data Registration Form

General Information			
Name	(Initials in Pinyin)	Hospital No.	
Gender	Male □ Female □	Age	y
Admission Date		ASA Grade	I □ II □ III □
Height	cm	Weight	kg
BMI		Phone number	
Education	Elementary School □ Junior High School □ High School □ University or Above □		
Work Status	Employed □ Unemployed □ Retired □	Smoking History	Yes □ No □
Living Situation	Independent Living □ Taken Care of by family □		
Diagnosis			
Planned Surgery			

Preoperative Medication Use			
Opioids	Yes □ No □	Benzodiazepines	Yes □ No □
Diuretics	Yes □ No □	ACEI/ARB	Yes □ No □
Digitalis drugs	Yes □ No □	β-blockers	Yes □ No □
Steroids	Yes □ No □	Calcium Channel Blockers	Yes □ No □
Anticoagulants	Yes □ No □	Nitrates	Yes □ No □
Antiplatelets	Yes □ No □	Antiarrhythmic Drugs	Yes □ No □
Lipid-lowering Drugs:	Yes □ No □	Hypoglycemic Drugs	Yes □ No □
Others		Drug Allergy History	

Preoperative Comorbidities and Examinations

Hypertension: Yes □ No □; Diabetes: Yes □ No □; Cardiovascular Disease: Yes □ No □;

Cerebrovascular Disease: Yes □ No □; Nervous System Disease: Yes □ No □; Liver Disease: Yes □ No □;

Kidney Disease: Yes □ No □; Immune Disease: Yes □ No □; Respiratory Disease: Yes □ No □; Tumor: Yes □ No □

Na+		Glu		Hct		Hb	
K+		BNP		Albumin		C-reactive protein	
BUN		Cr		WBC		Chest X-ray/CT	
ECG				Others			

**IV. Intraoperative Parameters**				
	Baseline	2 min after Induction	Surgery Start	Surgery End
Blood Pressure				
Heart Rate				
Sevoflurane (MAC)				
Intraoperative Events				
	Hypotension<br>MAP drop >30%	Bradycardia<br>HR <45 bpm	Hypertension<br>MAP rise >30%	Others
Occurrence Count				
Medication Dose				
	Propofol	Sufentanil	Dexmedetomidine	Remifentanil
Total Anesthetics				
	Ephedrine	Phenylephrine	Atropine	Others
Total Vasoactive Drugs				
Anesthesia and Surgery Time				

Induction Start Time:___________	Surgery Start Time: ___________
Surgery End Time: ___________	Anesthesia End Time:___________

**V. PACU Recovery Status**	
Time into PACU:______ Time out of PACU:______	PCA Pump Use Count:____________
15 min Post-Extubation LOS Score:______	15min Post-Extubation Pain Score:Resting__Coughing __

Hypotension: Yes □ No □	Nausea: Yes □ No □	Vomiting: Yes □ No □	Anti-PONV Medication: Yes□ No□

**VI. Postoperative Recovery Status**				
After operation	(1 h) Time:	(6 h)Time:	(24 h) Time:	(48 h) Time:
Visceral Pain (At Rest)				
Visceral Pain (Coughing)				
Incisional Pain (At Rest)				
Incisional Pain (Coughing)				
LOS Sedation Score				
48 Hours Complications				

Nausea: Yes □ No □	Vomiting: Yes □ No □	Antiemetic: Yes □ No □	Anti-emetic:_________

Pruritus:Yes □ No □	Respiratory Depression: Yes □ No □	Dizziness: Yes □ No □	Constipation:Yes □ No □
Others			
Postoperative 24 Hours Analgesic Dosage:_________		Postoperative 48 Hours Analgesic Dosage:_________	
Remedial Analgesia: Yes □ No □		Remedial Analgesia Details: _________	
Overall Satisfaction (Very Satisfied, Satisfied, Fair, Dissatisfied)		Postoperative flatus Time:____Hospitalization time:___	

Respiratory depression is defined as: decreased respiratory rate, decreased SPO2, need for assisted ventilation, or intubation.

**QoR-15**			
	Follow-up Time (Postoperative)		
How have you been feeling?	24 hour	48 hour	72 hour
(0 to 10, where: 0 = none of the time [poor] and 10 = all of the time [excellent])			
Able to breathe easily (0-10)			
Been able to enjoy food (0-10)			
Feeling rested (0-10)			
Have had a good sleep (0-10)			
Able to look after personal toilet and hygiene unaided (0-10)			
Able to communicate with family or friends (0-10)			
Getting support from hospital doctors and nurses (0-10)			
Able to return to work or usual home activities (0-10)			
Feeling comfortable and in control (0-10)			
Having a feeling of general well-being (0-10)			
Have you had any of the following?			
(10 to 0, where: 10 = none of the time [excellent] and 0 = all of the time [poor])			
Moderate pain (10-0)			
Severe pain (10-0)			
Nausea or vomiting (10-0)			
Feeling worried or anxious (10-0)			
Feeling sad or depressed (10-0)			

NRS Score：

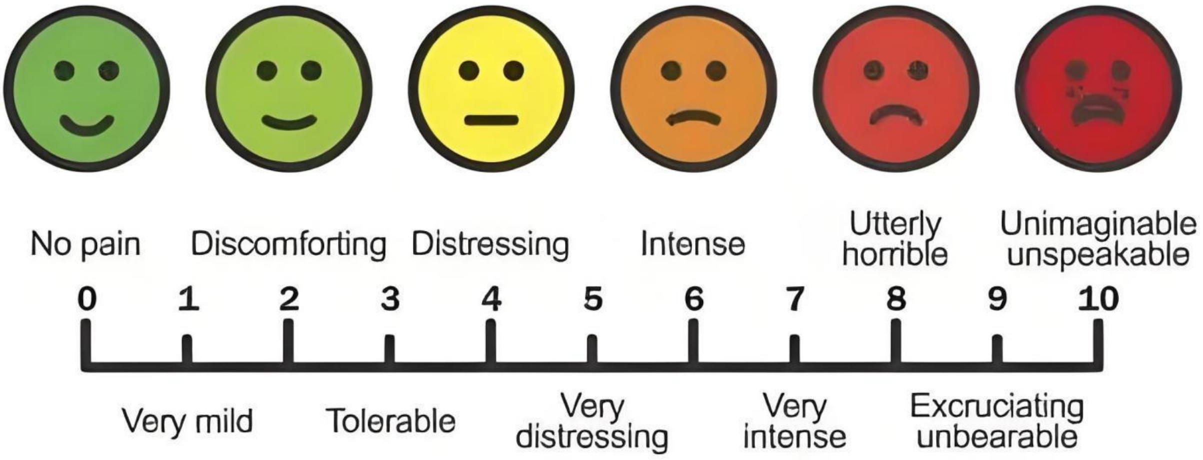

Level of Sedation (LOS) Scale:

0: Awake and alert

1: Some drowsy, easy to wake up

1S: Sleeping state, easy to wake up

2: Frequent drowsiness, easy to wake up, but unable to maintain a continuous of wakefulness

3: Hard to wake up and unable to stay in an awake state
