# Supplementary material for: Oxycodone vs. sufentanil combined with quadratus lumborum block vs. transverse abdominis plane block in laparoscopic major gastrointestinal surgery: A randomized factorial trial protocol
Source: Heliyon. 2024 Aug 15;10(16):e36186. doi: 10.1016/j.heliyon.2024.e36186 (PMC11381733; doi:10.1016/j.heliyon.2024.e36186)
Supplement: Multimedia component 2 [file mmc2.pdf]

苏州大学附属第一医院医学伦理委员会伦理审查批件

批件号：（2024）伦审批第 039 号

项目名称	羟考酮或舒芬太尼患者自控镇痛联合腰方肌或腹横肌平面阻滞对腹腔镜胃肠大手术后恢复的影响：一项随机、对照、析因设计临床试验		
研究类别	临床科研项目		
申办方/发起方	苏州大学附属第一医院麻醉科		
主要研究者	彭科	承担科室	麻醉科
伦理受理号	2024039	受理时间	2024-01-25
审查时间	2024-01-30	审查类别	初始审查
审查地点	总院综合楼 303 会议室	审查方式	会议审查
审查意见	依据我国相关法律、法规和国际伦理准则，伦理委员会对本项研究的研究方案、知情同意书、受试者招募材料及其他有关内容进行了会议审查，经投票表决，审查结果为同意开展本项研究。		
跟踪审查频率	本研究项目批准后 每 12 个月 向本伦理委员会递交研究进展报告。		

主任委员（签名）：陈罡

苏州大学附属第一医院医学伦理委员会（盖章）

日期：2024 年 1 月 31 日

（印章：苏州大学附属第一医院医学伦理委员会）

研究者/申办方须知：

1. 请遵循 NMPA/GCP 和《赫尔辛基宣言》的原则、遵循我国相关法律法规、遵循伦理委员会批准的方案开展研究，保护受试者的权益与安全。
2. 研究开始前，请申办者/研究者完成临床试验注册。
3. 研究过程中，请依规提交修正案申请，安全性报告，方案偏离报告，暂停/终止研究报告。
4. 根据伦理委员会批件中规定的年度/定期跟踪审查频率，无论研究开始与否，请在伦理跟踪审查到期前 1 个月提出跟踪审查的申请并递交研究进展报告。
5. 研究过程中，发生为消除对受试者紧急危害的研究方案的偏离或者修改；增加受试者风险或者显著影响临床研究实施的改变；所有可疑且非预期严重不良反应；可能对受试者的安全或者临床研究的实施产生不利影响的新信息，请及时报告伦理委员会。
6. 完成临床研究，须及时提交研究结题报告交伦理委员会审查。
7. 本批件有效期一年（自批准之日起）。

伦理委员会地址及联系方式	地址：江苏省苏州市姑苏区平海路 899 号苏大附一院总院综合楼 1312 办公室<br>电话：0512-67972861，0512-67972743；E-mail:sdfyec@163.com

扫描全能王 创建

审查文件清单

1.研究方案（版本号：1.0，日期：2023-08-01）

2.知情同意书（版本号：1.0，日期：2023-08-01）

3.病例报告表（版本号：1.0，日期：2023-08-01）

4.研究者简历

5.研究者声明

6.研究团队分工明细表

7.涉及人的遗传资源使用管理声明

8.研究经费来源及研究成果发布形式说明

9.利益冲突声明

扫描全能王 创建

苏州大学附属第一医院医学伦理委员会
会议签到表

会议时间	2024 年 01 月 30 日（星期二）下午 13:30-16:30
会议地点	苏州大学附属第一医院总院综合楼 303 会议室

姓　名	性别	委员会任职	职　称	专　业	工作单位	签　名
陈　罡	男	主任委员	主任医师	神经外科	苏州大学附属第一医院	
朱雪松	男	副主任委员	研究员	基础医学	苏州大学附属第一医院	
徐溢涛	男	副主任委员	副研究员	医院管理	苏州大学附属第一医院	
缪丽燕	女	委　员	主任药师	临床药学	苏州大学附属第一医院	
于树贵	男	委　员	教授	伦理学	苏州大学	
王进红	女	委　员	主任医师	内分泌科	苏州大学附属第一医院	
朱志伟	男	委　员	副教授	社会学	苏州大学	
张　华	男	委　员	主任药师	临床药学	苏州大学附属第一医院	
陈　成	男	委　员	主任医师	呼吸科	苏州大学附属第一医院	
汤在祥	男	委　员	教授	卫生统计学	苏州大学	
陈苏宁	男	委　员	主任医师	血液科	苏州大学附属第一医院	
张拥军	男	委　员	律　师	法学	北京隆安（苏州）律师事务所	
宋建平	女	委　员	主任医师	心血管内科	苏州大学附属第一医院	
唐晓文	女	委　员	主任医师	血液科	苏州大学附属第一医院	
章　斌	男	委　员	主任医师	核医学科	苏州大学附属第一医院	
韩　悦	女	委　员	主任医师	血液科	苏州大学附属第一医院	
魏雪栋	男	委　员	副主任医师	泌尿外科	苏州大学附属第一医院	

扫描全能王 创建
