# Supplementary material for: Oxycodone vs. sufentanil combined with quadratus lumborum block vs. transverse abdominis plane block in laparoscopic major gastrointestinal surgery: A randomized factorial trial protocol
Source: Heliyon. 2024 Aug 15;10(16):e36186. doi: 10.1016/j.heliyon.2024.e36186 (PMC11381733; doi:10.1016/j.heliyon.2024.e36186)
Supplement: Multimedia component 3 [file mmc3.pdf]

Subject Number: Version Number: 1.0

Version Date: August 1, 2023

Informed Consent Form

Oxycodone- vs. sufentanil-based patient-controlled analgesia combined with quadratus lumborum block vs. transverse abdominis plane block in laparoscopic major gastrointestinal surgery: a randomized factorial trial

Dear Madam/Sir:

You are invited to participate in a clinical study. The following paragraphs describe the background, purpose, methods, potential benefits and discomforts, inconveniences, and your rights related to this study. Please read carefully before participating. This Informed Consent Form provides information to help you decide whether to participate. If you have any questions, please ask the doctor in charge of the study to ensure you fully understand the content. Your participation is voluntary. If you agree to participate, please sign on the signature page of this Informed Consent Form.

I. Research Background

It is reported that the global burden of cancer is continuously increasing. Worldwide, an estimated 19.3 million new cancer cases and nearly 10 million cancer deaths occurred in 2020. The global cancer burden is expected to reach 28.4 million cases by 2040, a 47% increase from 2020, with stomach and colorectal cancers ranking high. Currently, gastrointestinal cancers are treated comprehensively, with surgical resection being the only cure. With the rise of fast-track surgery, laparoscopic minimally invasive surgery is more advocated. However, factors such as pneumoperitoneum pressure, increased operation time, and changes in body position still result in severe somatic and visceral pain for about 48 hours after surgery, affecting early postoperative activity and gastrointestinal motility, delaying recovery, extending hospital stays, and increasing medical costs. Effective intraoperative and postoperative analgesia can reduce postoperative complications, leading to faster

recovery, shorter hospital stays, and increased patient satisfaction. Sufentanil, as a classic opioid, is widely used in laparoscopic major gastrointestinal surgery, while oxycodone, a semi-synthetic opioid analgesic, has dual agonist effects on opioid μ and κ receptors, relieving both visceral and somatic pain. Transversus Abdominis Plane Block (TAPB) and Quadratus Lumborum Block (QLB) have been recognized and used in clinical studies for postoperative pain after abdominal surgery. Studies show that compared to TAPB, QLB can block both somatic and visceral pain, facilitating early postoperative recovery and movement. In laparoscopic major gastrointestinal surgery, the feasibility of intravenous oxycodone or sufentanil combined with TAPB or QLB for postoperative recovery quality evaluation requires further clinical research confirmation.

II. Study Name and Purpose

The name of this study is: "Oxycodone- vs. sufentanil-based patient-controlled analgesia combined with quadratus lumborum block vs. transverse abdominis plane block in laparoscopic major gastrointestinal surgery: a randomized factorial trial"

The purpose of this study is to explore which analgesic method, intravenous oxycodone or sufentanil patient-controlled analgesia combined with TAPB or QLB, can better improve the quality of recovery and promote early rehabilitation after laparoscopic major gastrointestinal surgery.

III. Research Methods and Content

This study will determine your eligibility based on your surgery type and entry criteria. You will be randomly assigned to one of four groups: Sufentanil with TAPB, Sufentanil with QLB, Oxycodone with TAPB, and Oxycodone with QLB, with 30 cases in each group. Using different analgesic modalities, we will collect medical information and data during your routine clinical anesthesia process and postoperative recovery. We will evaluate and analyze this medical information data. We will follow up with you from after surgery until discharge, asking and recording questions about your recovery, pain, and related complications.

IV. Study Process and Timeline

The entire study is expected to be completed within one year, with a total of 120 subjects planned. The study process consists of a screening period and a follow-up period. Your full participation in the study will take about 15 days, with no additional auxiliary examinations required.

V. Potential Benefits of Participating in the Study

Your participation may help alleviate postoperative pain. The information obtained from the study may lead to more measures and methods to improve the quality of postoperative recovery management, providing a theoretical basis and experience for future surgical patients' anesthetic management and more choices for improving postoperative recovery in patients undergoing laparoscopic major gastrointestinal surgery. Additionally, the cost of the study-related drug will be waived.

VI. Potential Risks and Discomforts of Participating in the Study

General anesthesia will be used in this study, with efforts to ensure a smooth process for the patient. The safety of general anesthesia is very high, but there are still potential risks during and shortly after the surgery: (1) respiratory depression, airway obstruction, aspiration or aspiration pneumonia, respiratory failure; (2) arrhythmias, hypotension, hypertension, heart failure; (3) tooth loss, hoarseness, laryngeal edema, postoperative pain, nausea, vomiting, agitation, delayed awakening, or awareness during surgery; (4) drug hypersensitivity or allergy, transfusion reactions; (5) water, electrolyte, and acid-base balance disorders, hemorrhagic shock; (6) central and peripheral nerve damage, postoperative headache, pulmonary complications. The anesthesiologist in charge or above will monitor the entire anesthesia process for safety. The anesthesiologist is fully responsible for monitoring and managing various conditions during the perioperative period according to regulations, routines, and standards. In case of deterioration, we will promptly inform the patient's family, and in the event of life-threatening situations, we guarantee full resuscitation efforts.

Please understand and support this. For more details, see the "Anesthesia Informed Consent" in the medical records, signed by the patient or authorized person.
The nerve block technique required for this study has been routinely performed in our department for many years, with rich clinical experience and no adverse events to date. In the event of a serious adverse reaction related to the study, appropriate treatment measures will be taken, and the Ethics Committee will be immediately notified, and economic compensation will be provided in accordance with Chinese laws and regulations.

VII. Treatment and Economic Compensation for Injuries Related to the Study

The intervention measures and other anesthetic drugs used in this study are all routine clinical anesthetic drugs and will not increase the risk beyond routine diagnosis and treatment for the subjects. If a subject suffers an injury related to the study, we will bear the related medical expenses and corresponding economic compensation according to relevant laws and regulations of our country.

VIII. Conventional Treatment Plans Outside of This Study

In addition to participating in this study, you can follow conventional postoperative pain management plans.

IX. Rights of the Subjects

This study will not cause harm to your body, mind, or social relationships, nor will it negatively affect the diagnosis and treatment of your condition. The entire research process is supervised by our hospital's Ethics Committee, and you can consult the research doctor for any questions during the study. Your participation is entirely voluntary; you can withdraw from the study at any time without reason, and it will not affect your relationship with medical staff or future treatment; all your personal information and observation records are confidential and used only for this study; during the trial, you can always learn about relevant information and materials, and you can contact the research doctor and team members at any time if there are

problems or questions.

X. Confidentiality of Clinical Research Data

Researchers are responsible for processing your research data in accordance with applicable data protection regulations. However, the Ethics Committee and higher administrative management departments can access these materials during inspections. Research results may be published in medical journals/conferences, but your identity will not be disclosed.

After signing this Informed Consent Form, it indicates that you agree to the collection, use, and sharing of your health information data by the research doctor and research center personnel. Your authorization for us to use your health information remains valid until the end of the study and before the results are determined. However, you can withdraw your Informed Consent at any time through the research doctor.

XI. Collection and Management of Human Biological Samples

Researchers declare that this study does not involve the collection and management of the subjects' biological samples.

XII. Contact Information

Before you sign this consent form, all members of the research team will answer all your questions. If you still have questions, suggestions, or opinions after signing this consent form, you can also communicate with the researcher. You can always learn about the information and progress of this study.

Researcher and contact information: Ke Peng, 15962155989, pengke0422@163.com

Ethics Committee contact and contact information: Shuangjie Wu, 0512-67972743

XIII. Declaration and Signature

Subject's declaration: I have carefully read this Informed Consent Form, I have had the opportunity to ask questions, and all questions have been answered. I understand that participation in this study is voluntary, I can choose not to participate in this study,

or I can withdraw from the study at any time without discrimination or retaliation, and my medical treatment and rights will not be affected.

If I need other treatments, or if I do not follow the research plan, or for any other reasonable reason, the research doctor can terminate my continued participation in this clinical study.

I voluntarily agree to participate in this clinical study, and I will receive a copy of the signed "Informed Consent Form"

Subject's name:________________ Subject's signature:________________

Date:___________________ Mobile phone number:__________________

Legal representative's name:______Legal representative's signature:______

Date:___________________ Mobile phone number:__________________

Relationship to the subject:___________

Reason why the subject cannot sign the Informed Consent Form:__________

Researcher's declaration: I have accurately informed the subject of the content of the Informed Consent Form and answered the subject's questions. The subject voluntarily participates in this clinical study.

Researcher's name: ____________ Researcher's signature:______________

Date:___________________ Mobile phone number:___________________
