# Supplementary material for: Oxycodone vs. sufentanil combined with quadratus lumborum block vs. transverse abdominis plane block in laparoscopic major gastrointestinal surgery: A randomized factorial trial protocol
Source: Heliyon. 2024 Aug 15;10(16):e36186. doi: 10.1016/j.heliyon.2024.e36186 (PMC11381733; doi:10.1016/j.heliyon.2024.e36186)
Supplement: Multimedia component 4 [file mmc4.pdf]

Research Protocol

Version Number: 1.0

Date: August 1, 2023

I. Study Title

Oxycodone- vs. sufentanil-based patient-controlled analgesia combined with quadratus lumborum block vs. transverse abdominis plane block in laparoscopic major gastrointestinal surgery: a randomized factorial trial protocol

II. Research Background

It is reported that the global burden of cancer is continuously increasing. Worldwide, an estimated 19.3 million new cancer cases and nearly 10 million cancer deaths occurred in 2020. The global cancer burden is expected to reach 28.4 million cases by 2040, a 47% increase from 2020, with stomach and colorectal cancers ranking high, posing more challenges and demands on society and healthcare work [1]. Currently, gastrointestinal cancers are treated comprehensively, with surgical resection being the only cure [2]. With the rise of Enhanced Recovery After Surgery (ERAS) [3], laparoscopic minimally invasive surgery is more advocated. However, factors such as pneumoperitoneum pressure, increased operation time, and changes in body position still result in severe somatic and visceral pain for about 48 hours after surgery, affecting early postoperative activity and gastrointestinal motility, delaying recovery, extending hospital stays, and increasing medical costs. Effective intraoperative and postoperative analgesia can reduce postoperative complications, leading to faster recovery, shorter hospital stays, and increased patient satisfaction. Single analgesic drugs have significant side effects with large dosages, and multimodal analgesia is gradually being promoted.

Sufentanil, as a classic opioid, is widely used in laparoscopic major gastrointestinal surgery, while oxycodone, a semi-synthetic opioid analgesic, has dual agonist effects on opioid μ and κ receptors, relieving both visceral and somatic pain [4]. Transversus Abdominis Plane Block (TAPB) and Quadratus Lumborum Block (QLB) have been recognized and used in clinical studies for postoperative pain after abdominal surgery [5, 6]. Studies show that compared to TAPB, QLB can block both somatic and visceral pain, facilitating early postoperative recovery and movement [7, 8]. In laparoscopic major gastrointestinal surgery, the feasibility of intravenous oxycodone or

sufentanil combined with TAPB or QLB for postoperative recovery quality evaluation requires further clinical research confirmation.

III. Research Objective

Compared with patient-controlled analgesia with intravenous oxycodone or sufentanil combined with lumbar quadratus block or transverse abdominal muscle block, to explore the best multi-mode analgesia scheme to improve the quality of recovery after laparoscopic gastrointestinal surgery and promote the early rehabilitation of patients.

IV. Study Design (including overall design, sample size, number of participating units, study steps, and study timeline)

1. Overall Design

This study is a prospective, single-center, randomized, subject-blind, evaluator-blind, controlled, factorial, and efficacy-designed clinical study conducted by researchers. 120 patients undergoing laparoscopic major gastrointestinal surgery from March 2024 to March 2025 will be enrolled in the study (oxycodone plus QLB group, oxycodone plus TAPB group, sufentanil plus QLB group, sufentanil plus TAPB group, n = 30).

2. Sample Size Calculation

The sample size for this trial is based on the main effects of oxycodone and QLB. According to our preliminary trial results, the QoR-15 score at 24 hours after laparoscopic major gastrointestinal surgery in the sufentanil combined with TAPB group was 99±10. We expect that the use of oxycodone or QLB can improve the QoR-15 score by 6 points (based on literature, this threshold is clinically significant), with α=0.025 and a power of 80%, 55 patients are needed per group. Considering possible loss to follow-up, it is planned to enroll 60 cases per group for oxycodone and sufentanil (including those using TAPB or QLB); TAPB and QLB groups will also enroll 60 cases each (including the use of oxycodone or sufentanil). Therefore, this study plans to finally include a total of 120 cases, N1=N2=N3=N4=30 (statistical software: PASS 15, NCSS, LLC. Kaysville, Utah, USA).

3. Participating Units

The First Affiliated Hospital of Soochow University

4. Ethics and Registration

After obtaining ethical approval, the study will be registered online at the Chinese Clinical Trial Registration Center, a primary registrant of WHO, and obtain a clinical trial registration number. Clinical trial registration is completed before the enrollment of the first patient. All enrolled patients are fully informed about the process of this study and sign an informed consent form.

5. Study Steps and Timeline

After ethical registration, the initial screening of patients will begin according to the plan, sign the informed consent form, enroll according to the random list generated by the computer, implement anesthesia and surgery, and visit at the specified time points after surgery until the patient is discharged. This study is expected to be completed within one year.

V. Study Population (including inclusion criteria, exclusion criteria, withdrawal criteria, and termination criteria)

1. Inclusion Criteria

Age≥18 years, gender not limited; American Society of Anesthesiologists (ASA) I-III class; patients planned to undergo laparoscopic major gastrointestinal surgery under general anesthesia (defined as surgery time≥2 hours, involving partial resection of the stomach or intestine); clearly understand the trial process and voluntarily participate in this study, sign the informed consent form.

2. Exclusion Criteria

Non-planned or emergency surgery; BMI≥35 kg/m^2; allergy to drugs in the study; severe cardiopulmonary disease (myocardial infarction, heart failure, respiratory failure); severe cerebrovascular disease (cerebral hemorrhage, stroke); severe liver and kidney diseases (Child-Pugh class C, renal replacement therapy); severe neurological diseases (Parkinson's disease, Alzheimer's disease), antipsychotic drugs, alcoholism, long-term use of opioids or other analgesics; hearing or language barriers that prevent communication, unwilling to use postoperative intravenous analgesia (PCIA), unable to understand postoperative recovery scales, pain score scales.

3. Withdrawal Criteria

If the subject has significant organ dysfunction, drug allergy, poor compliance, disease progression, or severe adverse reactions requiring cessation of trial medication or other treatment methods during the trial, the researcher will withdraw the subject from the trial; or if the subject wishes to adopt other treatment methods or actively withdraws from the trial for no reason.

4. Termination Criteria

The Ethics Committee supervises the trial throughout the process. If the researcher does not follow the approved plan or relevant regulations to conduct the clinical trial, it can be terminated.

VI. Intervention Plan for Study Implementation

1. Pre-Anesthetic Preparation

Patients are visited by independent anaesthesia nurses or anesthesiologists who does not participate in the follow-up study one day before operation, the patients will be screened according to the inclusion criteria and exclusion criteria, and the informed consent of anesthesia and the research project is signed. No preoperative medication will be used, fasting for at least 6 hours and drinking for 2 hours before operation.

2. Anesthetic Monitoring

Standard monitoring after the patient enters the operating room includes: electrocardiogram (ECG), pulse oximetry (SpO2), bispectral index (BIS), non-invasive blood pressure (NIBP), radial artery puncture to monitor invasive blood pressure, and monitoring body temperature (nasopharyngeal temperature).

3. Anesthetic Induction and Maintenance

After preoxygenation via a mask with 5L/min of pure oxygen, anesthetic induction is initiated with propofol at a dose of 1.5-2mg/kg, sufentanil at 0.2μg/kg, and cisatracurium at 0.2mg/kg for endotracheal intubation. Following intubation, the patient is connected to a ventilator for controlled breathing with an inspired oxygen concentration of 60%, in volume control mode, a tidal volume of 6-8ml/kg, a frequency of 12-15 breaths per minute, an inspiratory to expiratory ratio of 1:2, maintaining end-tidal carbon dioxide (PetCO2) between 35-40mmHg. Anesthesia depth is maintained with sevoflurane inhalation, continuous infusion of dexmedetomidine, remifentanil, and cisatracurium. The depth of anesthesia is monitored with bispectral index (BIS) values kept

between 40-60, or by measuring the minimum alveolar concentration (MAC) of the expired gas at 0.6-1.3. Prior to induction, 5mg of dexamethasone and 0.075mg of palonosetron are administered to prevent postoperative nausea and vomiting (PONV). When rinsing the abdominal cavity in preparation for drain placement, the infusion of dexmedetomidine and cisatracurium is halted, and 50mg of flurbiprofen axetil is given. Depending on the study group assignment, a single dose of oxycodone or sufentanil is administered. At the end of the skin closure, the infusion of remifentanil is discontinued, and a patient-controlled intravenous analgesia (PCIA) device based on oxycodone or sufentanil is connected (background dose of 1ml/h, with a single bolus dose of 2mL and a lock time of 5 minutes).

4. Intervention Measures

Oxycodone combined with QLB group (before surgery: sufentanil 0.2μg/kg IV; under ultrasound guidance in the supine position, 0.375% ropivacaine 40ml is administered for bilateral QLB, and 15 minutes before the end of surgery, oxycodone 0.2 mg/kg IV is given).

Oxycodone combined with TAPB group (before surgery: sufentanil 0.2μg/kg IV; under ultrasound guidance in the supine position, 0.375% ropivacaine 40ml is administered for bilateral TAPB, and 15 minutes before the end of surgery, oxycodone 0.2 mg/kg IV is given).

Sufentanil combined with QLB group (before surgery: sufentanil 0.2μg/kg IV; under ultrasound guidance in the supine position, 0.375% ropivacaine 40ml is administered for bilateral QLB, and 15 minutes before the end of surgery, sufentanil 0.2μg/kg IV is given).

Sufentanil combined with TAPB group (before surgery: sufentanil 0.2μg/kg IV; under ultrasound guidance in the supine position, 0.375% ropivacaine 40ml is administered for bilateral TAPB, and 15 minutes before the end of surgery, sufentanil 0.2μg/kg IV is given).

Note: For the oxycodone group, a PCIA containing 100mg of oxycodone is used; for the sufentanil group, a PCIA containing 100μg of sufentanil is used, both until 48 hours after operation.

VII. Observation indicators of the study

1. Primary study outcome: QoR-15 recovery quality score at 24 hours after operation.

2. Secondary study outcomes:

(1) Visceral pain score at rest and cough at 1, 6, 24, and 48 hours after operation;

(2) Incision pain score at rest and cough at 1, 6, 24, and 48 hours after operation;

(3) Consumption of analgesics at 0-24 h and 24-48 h after operation (equivalent sufentanil, i.e. oxycodone 1 mg = sufentanil 1 μ g);

(4) QoR-15 recovery score at 48 and 72 hours after operation;

(5) The need for rescue analgesia and the amount of analgesic medication used within 24 and 48 hours after operation;

(6) Time to first flatus after operation;

(7) Time to discharge after operation;

(8) Events of hypotension, hypertension, bradycardia, and tachycardia;

(9) Sedation scores at 1, 6, 24, and 48 hours after operation;

(10) Occurrence of nausea, vomiting, use of antiemetic medication, respiratory depression, delirium, and dizziness within 48 hours after operation.

3. Other non-outcome indicators:

Blood pressure and heart rate at admission, 2 minutes after anesthesia induction, at the start of surgery, and at the end of surgery; dosage of anesthetic and vasoactive medications; duration of surgery and anesthesia.

VIII. Study Follow-up plan

All patients will have follow-up visits at 1, 6, 24, and 48 hours postoperatively, as well as at the time of discharge.

IX. Safety Monitoring and Management Plan for Adverse Events

The intervention measures and other anesthetic drugs used in this study are all anesthetic medications and methods routinely used in clinical practice and will not pose risks to the subjects beyond those of conventional diagnosis and treatment. During surgery, the entire anesthetic process will be monitored for safety by anesthesiologists at the level of attending physician or above. Possible adverse reactions and treatment measures are as follows: Failure of ultrasound-guided nerve block procedure, exclusion of the case from the study; Hypertension (an increase in MAP exceeding 30% of the baseline value) and tachycardia (HR > 100 beats/min), in the case of adequate anesthetic depth, intravenous administration of Perdipin 0.5mg per dose or Esmolol 20mg per dose; Hypotension (a decrease in MAP exceeding 30% of the baseline value), intravenous fluid

infusion, injection of Ephedrine 6mg per dose or Phenylephrine 25-50ug per dose; Bradycardia (HR < 45 beats/min), intravenous injection of Atropine 0.5mg per dose. Postoperative pain rescue treatment plan: If the patient's NRS score is 4 or higher, Flurbiprofen Axetil 50mg will be administered intravenously, which may be repeated once within 24 hours if necessary. Postoperative nausea and vomiting rescue treatment plan: If the patient experiences nausea and vomiting after surgery, treatments such as Palonosetron or Metoclopramide may be administered as appropriate. Record any adverse events, including type, time, duration, and management method; continue to follow up until the issue is fully resolved or treatment is terminated. If any serious adverse events occur, the study protocol will be stopped immediately and treatment will commence; if the attending anesthesiologist or principal investigator deems it necessary, the study protocol may be temporarily or permanently halted, and the time and reason for the study interruption will be recorded in the Case Report Form (CRF); for any serious adverse events, in addition to the active treatment and recording mentioned above, a written report will be notified to the principal investigator and the ethics committee within 24 hours.

X. Management and Statistical Analysis

All data will be recorded by independent researchers who are unaware of the study group allocation in the Case Report Form (CRF), and then uploaded to the electronic network database (https://www.91trial.com). The principal investigator will ensure the accuracy and completeness of the data, and the collected data will be supervised. The SPSS software will be used, employing the Kolmogorov-Smirnov test to assess whether continuous variables are normally distributed. Quantitative data such as age, BMI, operation time, anesthesia time, HR, MAP, etc., will be represented by mean ± standard deviation (X±S), and intergroup comparison will be made using the independent sample t-test. Non-normally distributed variables, such as NRS scores, the use of Ephedrine or Atropine, and PONV with postoperative pain rescue treatment, will be represented by median (interquartile range [IQR]) and compared using the rank-sum test. Categorical data such as gender, ASA classification, etc., will be represented by the number of cases and percentage (%), and intergroup comparison will be made using the chi-square test. Data at different time points will be analyzed using repeated measures analysis of variance. A difference is considered statistically significant if $P < 0.05$.

XI. References

[1] H. Sung, J. Ferlay, R.L. Siegel, M. Laversanne, I. Soerjomataram, A. Jemal, F. Bray, Global Cancer Statistics 2020: GLOBOCAN Estimates of Incidence and Mortality Worldwide for 36 Cancers in 185 Countries, CA: a cancer journal for clinicians 71(3) (2021) 209-249.

[2] F.H. Wang, L. Shen, J. Li, Z.W. Zhou, H. Liang, X.T. Zhang, L. Tang, Y. Xin, J. Jin, Y.J. Zhang, X.L. Yuan, T.S. Liu, G.X. Li, Q. Wu, H.M. Xu, J.F. Ji, Y.F. Li, X. Wang, S. Yu, H. Liu, W.L. Guan, R.H. Xu, The Chinese Society of Clinical Oncology (CSCO): clinical guidelines for the diagnosis and treatment of gastric cancer, Cancer communications (London, England) 39(1) (2019) 10.

[3] S.H. Kang, Y. Lee, S.H. Min, Y.S. Park, S.H. Ahn, D.J. Park, H.H. Kim, Multimodal Enhanced Recovery After Surgery (ERAS) Program is the Optimal Perioperative Care in Patients Undergoing Totally Laparoscopic Distal Gastrectomy for Gastric Cancer: A Prospective, Randomized, Clinical Trial, Annals of surgical oncology 25(11) (2018) 3231-3238.

[4] C. Staahl, L. Christrup, S. Andersen, L. Arendt-Nielsen, A. Drewes, A comparative study of oxycodone and morphine in a multi-modal, tissue-differentiated experimental pain model, Pain 123 (2006) 28-36.

[5] K. Tanggaard, R.P. Hasselager, E.R. Hølmich, C. Hansen, M. Dam, T.D. Poulsen, F. Bærentzen, J.R. Eriksen, I. Gögenur, J. Børglum, Anterior quadratus lumborum block does not reduce postoperative opioid consumption following laparoscopic hemicolectomy: a randomized, double-blind, controlled trial in an ERAS setting, Reg Anesth Pain Med (2022).

[6] S. Charlton, A.M. Cyna, P. Middleton, J.D. Griffiths, Perioperative transversus abdominis plane (TAP) blocks for analgesia after abdominal surgery, The Cochrane database of systematic reviews (12) (2010) Cd007705.

[7] R. Blanco, T. Ansari, W. Riad, N. Shetty, Quadratus Lumborum Block Versus Transversus Abdominis Plane Block for Postoperative Pain After Cesarean Delivery: A Randomized Controlled Trial, Regional anesthesia and pain medicine 41(6) (2016) 757-762.

[8] G. Öksüz, B. Bilal, Y. Gürkan, A. Urfalioğlu, M. Arslan, G. Gişi, H. Öksüz, Quadratus Lumborum Block Versus Transversus Abdominis Plane Block in Children Undergoing Low Abdominal Surgery: A Randomized Controlled Trial, Regional anesthesia and pain medicine 42(5) (2017) 674-679.
